# Structural characterization of the late competence protein ComFB from *Bacillus subtilis*

**DOI:** 10.1042/BSR20140174

**Published:** 2015-03-31

**Authors:** Tatyana A. Sysoeva, Lukas B. Bane, Daphne Y. Xiao, Baundauna Bose, Scott S. Chilton, Rachelle Gaudet, Briana M. Burton

**Affiliations:** *Department of Molecular and Cellular Biology, Harvard University, Cambridge, MA 02138, U.S.A.

**Keywords:** *comF* operon, late competence operon, DNA uptake, natural transformation, competent *Bacillus subtilis*, BME, β-mercaptoethanol, cfu, colony forming unit, Com, genetic competence, CV, column volume, LB, lysogeny broth, NTPase, nucleotide triphosphate hydrolase, ORF, open reading frame, PABPC, poly(A)-binding protein homologue C-terminal domain, SEC, size exclusion chromatography, SeMet–ComFB, selenomethionine-substituted ComFB, UbCUE, ubiquitin-binding CUE domain

## Abstract

Many bacteria take up DNA from their environment as part of the process of natural transformation. DNA uptake allows microorganisms to gain genetic diversity and can lead to the spread of antibiotic resistance or virulence genes within a microbial population. Development of genetic competence (Com) in *Bacillus subtilis* is a highly regulated process that culminates in expression of several late competence genes and formation of the DNA uptake apparatus. The late competence operon *comF* encodes a small protein of unknown function, ComFB. To gain insight into the function of ComFB, we determined its 3D structure via X-ray crystallography. ComFB is a dimer and each subunit consists of four α-helices connected by short loops and one extended β-strand-like stretch. Each subunit contains one zinc-binding site formed by four cysteines, which are unusually spaced in the primary sequence. Using structure- and bioinformatics-guided substitutions we analyzed the inter-subunit interface of the ComFB dimer. Based on these analyses, we conclude that ComFB is an obligate dimer. We also characterized ComFB *in vivo* and found that this protein is produced in competent cells and is localized to the cytosol. Consistent with previous reports, we showed that deletion of ComFB does not affect DNA uptake function. Combining our results, we conclude that ComFB is unlikely to be a part of the DNA uptake machinery under tested conditions and instead may have a regulatory function.

## Abstract 2

Development of genetic competence in *Bacillus subtilis* results in expression of late competence genes, including *comFB*. *In vitro* and *in vivo* studies revealed that ComFB dimerizes, binds zinc, and possibly plays a regulatory role in competence development.

## INTRODUCTION

Many bacteria exhibit the ability to internalize exogenous DNA from their environment during the process of natural transformation. Such DNA uptake can facilitate DNA repair and is a major source for horizontal gene transfer leading to increased genetic diversity. Natural transformation promotes the spread of genes, including those related to antibiotic resistance and virulence among microbiological populations. This spread of antibiotic resistance poses a significant threat to modern human populations and therefore it is important to understand how bacteria take up DNA [1].

**Figure 1:**
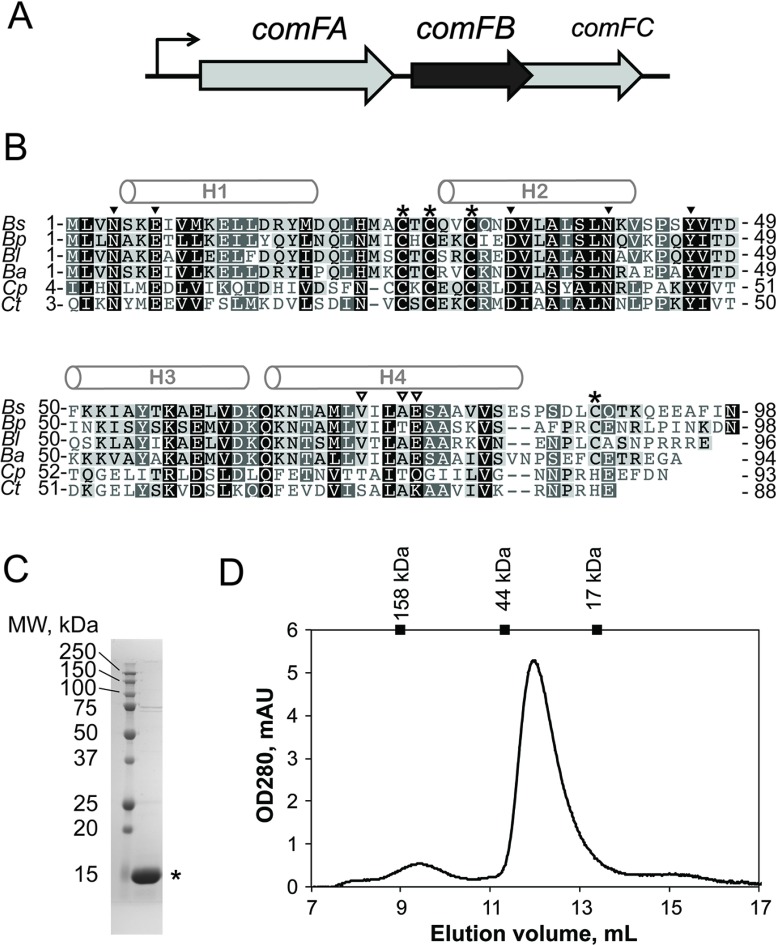
Late competence protein ComFB (**A**) *comF* operon in *B. subtilis* encodes for three proteins: ComFA, a DNA-helicase and two proteins of unknown function, ComFB and ComFC. The second and third ORFs overlap by four nucleotides. (**B**) Alignment of ComFB proteins from several firmicute species. α-Helices of *B. subtilis* ComFB fold are labeled. Cysteine residues are denoted with asterisks. Residues of the alternative dimerization interfaces, found in crystals, are marked with closed and open arrowheads respectively. *Ba*, *Bacillus amyloliquefaciens*; *Bl*, *Bacillus lichenomorphis*; *Bp*, *Bacillus pumilis; Bs*, *Bacillus subtilis*; *Cp*, *Clostridium phytofermentans*; *Ct*, *Clostridium thermocellum*. (**C**) SDS-PAGE analysis of the purified histidine–tagged ComFB protein. (**D**) Recombinant ComFB protein dimerizes in solution. ComFB protein was analyzed via SEC on a Superdex75 10/300 column. Molecular weight of purified ComFB monomer is 13.5 kDa. SEC peak position corresponds to ∼35 kDa as defined by calibration with the SEC standards of known molecular weight (indicated on the top bar of the graph).

The development of genetic competence (Com), the state in which a bacterial cell can take up exogenous DNA, is well-studied in *Bacillus subtilis*. In this bacterium, competence is traditionally induced by starving the cells of critical nutrients [2,3]. Only a subset of the bacterial population (∼10%–20% of the cells) will undergo a multistep process that ultimately results in the expression and assembly of the functional DNA uptake machinery necessary for competence. The process of differentiation of *B. subtilis* cells into genetically competent ones is highly interwoven with other developmental cell processes, such as entrance into sporulation or release of degradative enzymes. Development of competence depends on the accumulation of the master-regulator ComK. ComK protein is a transcription regulator that modifies the expression levels of more than 100 different genes, including a positive feedback loop that upregulates its own expression [4–6]. ComK upregulates the expression of the components of the DNA uptake apparatus encoded in several late *com* operons: *comC, comE, comG, comF*. The *comF* operon encodes three proteins: ComFA, a DNA helicase and two proteins of unknown function, ComFB and ComFC (Figure 1A) [7]. In the prior work, ComFA and ComFC were demonstrated to be important for the DNA uptake process; deletion of ComFA resulted in a decrease in transformation efficiency by three orders of magnitude. Moreover, single amino acid substitutions within the ComFA nucleotide triphosphate hydrolase (NTPase)-active site phenocopied the *comFA* deletion in its effect on genetic transformation, demonstrating that the NTPase activity is crucial to ComFA function [8]. A transposon insertion near the end of *comFB* was reported to decrease transformation efficiency ten fold [7]. Two ComFC homologues, ComF (Slr0388) from *Synechocystis* sp. strain PCC 6803 and open reading frame 2 (ORF 2) in the *Haemophilus influenzae com*101A locus, were also reported to be involved in transformation based on functional assays [9,10]. ComFC-like proteins contain a phosphoribosyltransferase (PRT) domain that may be involved in nucleotide salvaging upon DNA strand degradation [9] but their enzymatic activity and specificity has yet to be assessed.

To date, information regarding the role of ComFB is even more limited than that about the other two *comF* genes' products. Sequence analyses of the genomes of some studied competent bacterial organisms reveal that ComFB is not conserved in all species. Although ComFB is conserved in some species closely related to *B. subtilis* (Figure 1B), the *comF* operon encodes only the ComFA and ComFC homologues in other closely related organisms, such as *Bacillus anthracis* and *Bacillus cereus* [11].

Fluorescence microscopy analyses of the DNA uptake machinery in *B. subtilis* suggest that many late competence components localize to the cell poles [12–14]. ComFB fused with fluorescent protein appears to accumulate at the poles along with other late competence gene products and thus it was proposed that ComFB is a part of the DNA uptake machinery [14].

Previous work by Ogura [15] revealed that zinc uptake and homoeostasis in *B. subtilis* affect natural transformation and expression of *comF* operon [15]. Both the high affinity ZnuABC transporter and the low affinity transporter ZosA are required for the development of full competence. Decreased transformation efficiency in strains with ZosA mutations can be complemented with a high zinc concentration in the medium. Moreover, the ZnuA mutation results in specific down-regulation of *comF* operon transcription without an effect on other late competence operons. Again, the inhibition can be alleviated by addition of an increased amount of zinc salts in the growth medium.

To gain insight about the possible functions of the second protein encoded in the *comF* operon, ComFB, we investigated this protein *in vivo* and generated pure protein to determine its *in vitro* oligomeric state and high-resolution 3D structure. We find that ComFB is an obligate dimer with a dimerization interface highly conserved across a variety of bacterial species. ComFB tightly binds one zinc ion per subunit using an unusually-spaced four-cysteine motif. Although ComFB is clearly expressed in the cytoplasm of naturally competent cells, deletion of the *comFB* ORF does not have consequence on transformation efficiency. These findings provide a framework for further biochemical and functional analyses to investigate the physiological role of ComFB in naturally transformable organisms.

## EXPERIMENTAL

### Construction of *Escherichia coli* and *B. subtilis* strains

*Escherichia coli* and *B. subtilis* strains were maintained at 37°C in lysogeny broth (LB) medium (10 g·l^−1^ tryptone, 5 g·l^−1^ yeast extract, 5 g·l^−1^ NaCl) or on LB plates containing 1.5% Bacto agar. When appropriate, antibiotics were included in the growth medium as follows: 100 μg·ml^−1^ spectinomycin, 5 μg·ml^−1^ chloramphenicol, 5 or 50 μg·ml^−1^ kanamycin (Kan5 or Kan50) and 1 μg·ml^−1^ erythromycin plus 25 μg·ml^−1^ lincomycin (MLS). DSM agar plates [16] were used when plating for cfu (colony forming unit).

General methods for molecular cloning and construction of *E. coli* and *B. subtilis* strains were performed according to published protocols [16,17]. Chromosomal DNA isolated from *B. subtilis* PY79 was used as a template for PCR amplifications, unless otherwise specified. DNA was introduced into *B. subtilis* PY79 derivatives by transformation [18]. Bacterial strains used in the present study are summarized in Supplementary Table S1.

### Cloning and site-directed mutagenesis

Site-specific mutagenesis to obtain ComFB mutants was conducted using standard protocols using Pfu polymerase and two oligonucleotides for each mutation (oTS387/oTS388; oTS397/oTS398; oTS419/oTS420). Plasmid construction is detailed in the Supplemental Materials. Oligonucleotide sequences and are provided in Supplementary Table S2. All constructs were confirmed by sequencing.

### ComFB protein expression and purification

For protein expression, wild-type or mutant ComFB expression plasmids were transformed into the *E. coli* BL21(DE3) strain. A 5 ml overnight culture was inoculated into 1 l LB/Kan50 at a starting *A*_600_ of 0.01 and grown at 37°C. ComFB expression was induced at *A*_600_=0.4–0.6 with addition of 0.5 mM IPTG and with following overnight growth at 16°C. Cells were harvested by centrifugation (3000 ***g***) and resuspended in 25 ml lysis buffer [50 mM Hepes (pH 8.0), 300 mM NaCl, 2 mM β-mercaptoethanol (BME), 0.1 mM PMSF] and lyzed by passing twice through a Constant Systems cell disrupter at 17.5 kpsi. The cell lysate was clarified by centrifugation at 100000 ***g*** for 1 h using Ti70 rotor.

The clarified lysate was then applied to a 5 ml His60 Ni Superflow (Clontech) column equilibrated with Ni-binding buffer [50 mM Hepes (pH 8.0), 300 mM NaCl, 2 mM BME, 5% glycerol]. After washing with 10 column volume (CV) of Ni-wash buffer (Ni-binding buffer with 20 mM imidazole), the protein was eluted with 10-CV of Ni-elution buffer (Ni-binding buffer with 250 mM imidazole). Fractions containing ComFB were pooled and exchanged into a low-salt buffer [12.5 mM Hepes (pH 8.0), 75 mM NaCl, 2 mM BME, 5% glycerol] using a series of three 5-ml HiTrap Desalt (GE Healthcare Life Sciences) columns. The pH was then lowered from pH 8.0 to 6.0 by drop-wise addition of the protein solution into SP-binding buffer [50 mM BisTris (pH 6.0), 50 mM NaCl, 2 mM BME, 5% glycerol], with a final dilution factor of 1:5. The mixture was centrifuged at 100000 ***g*** for 30 min to remove any precipitated protein. The supernatant was then applied to a 5 ml HiTrap SP Sepharose HP (GE Healthcare Life Sciences) column equilibrated with SP-binding buffer. Two populations of His6–ComFB could be separated by a linear gradient of SP-elution buffer (SP-binding buffer with 1 M NaCl). A five-CV linear gradient up to 35% SP-elution buffer separated impurities from a first population of ComFB, which began eluting at 35% of elution buffer. After a five-CV wash at 35% elution buffer, an additional linear gradient from 35% to 65% SP-elution buffer served to elute a second population of ComFB. The molecular difference was analyzed by MS (see below) showing that the first fraction contains a non-specific modification. Because of the modification, only the second population was used in all subsequent experiments. A yield of 12 mg of wild-type ComFB was obtained from 1 l of culture.

### SDS-PAGE and Western blot analyses

Proteins were separated by SDS-PAGE and transferred to a nitrocellulose membrane (Bio-Rad) for immunoblot analysis. Membranes were probed with anti-GFP antibodies (1:10000; custom polyclonal serum, Covance). Peroxidase-conjugated goat anti-rabbit (1:10000, Jackson ImmunoResearch Laboratories) secondary antibodies were detected by chemiluminescence using Western Lightning reagent (PerkinElmer). Blots were imaged via ChemiDocXRS (Bio-Rad) and processed in QuantityOne (Bio-Rad).

### Mass spectrometric analyses of ComFB protein preparations

In developing the purification strategy for the protein, we noted that the protein elutes in two peaks from the anion exchange SP column. As both the protein fractions ran identically on SDS-PAGE, we used LC–ESI MS to investigate any chemical differences between the fractions. We found that the first methionine of ComFB is removed in both fractions, as expected. We also found the first peak from the SP column contained a species ∼256 Da heavier than predicted molecular weight of the protein. A search of a database for post-translational protein modifications (http://www.abrf.org/index.cfm/dm.home) suggested a phospho-glucose modification of the histidine–tag as a potential source of the mass difference [19].

### Selenium-substituted ComFB preparation

Selenomethionine-substituted ComFB (SeMet–ComFB) protein was obtained by overexpression in BL21(DE3) in defined rich media [20] with slight modifications. SeMet–ComFB was purified similarly to wild-type protein albeit with a lower yield of about 2 mg/l of culture. LC–ESI MS confirmed that a majority of the SeMet–ComFB protein indeed contained six selenium atoms out of a possible total of six (not including the cleaved N-terminal methionine residue).

### Crystallization and X-Ray diffraction data collection

Crystals were grown overnight by sitting drop vapour diffusion at 25°C by mixing 1 μl of 10 mg/ml protein with 0.5 μl of reservoir solution (0.2 M NaCl; 0.1 M BisTris, pH 5.5; 22% PEG 3350). SeMet–ComFB was more prone to precipitation but yielded crystals in the same conditions from lower protein concentrations (∼0.5–1 mg/ml). Crystals were soaked in cryoprotectant solution (reservoir solution with 25% glycerol) for ∼1 min before harvesting directly from the crystallization drop using a nylon loop. All crystals were cryo-cooled in liquid nitrogen. Diffraction data were collected at the Advanced Photon Source using either the ID-E or ID-C beamline and processed with HKL2000 [21]. Native ComFB crystals were in space group P2_1_ with cell dimensions of *a*=41.5 Å (1 Å=0.1 nm), *b*=123.6 Å, *c*=41.8 Å; *α*=90°, *β*=93.9°, *γ*=90° and SeMet–ComFB–P22_1_2_1_ with cell dimensions *a*=40.7 Å, *b*=41.1 Å, *c*=123.0 Å (Table 1).

**Table 1:** Data collection and refinement statistics Statistics for the highest-resolution shell are shown in parentheses; Abbreviation: RMS, root mean square deviation.

	SeMet–ComFB	Native ComFB	Zn^2+^ ComFB
**Data collection**			
Wavelength (Å)	0.9792	0.9792	1.2782
Resolution range (Å)	40.72–2.746 (2.84–2.746)	34.54–2.427 (2.513–2.427)	50.0–2.71 (2.76–2.71)
Space group	P22_1_2_1_	P2_1_	P22_1_2_1_
Unit cell (*a, b, c, α, β, γ*)	40.7, 41.1, 123.0, 90°, 90°, 90°	41.5, 123.56, 41.8, 90°, 93.9°, 90°	38.04, 38.68, 120.21, 90°, 90°, 90°
Total reflections	34931 (2677)	45062 (3301)	57071 (2118)
Unique reflections	5768 (529)	14767 (1102)	5210 (238)
Multiplicity	6.1 (5.1)	3.1 (3.0)	11.0 (8.9)
Completeness (%)	99.59 (97.24)	93.00 (69.84)	99.80 (99.60)
Mean I/*σ*(I)	14.37 (2.41)	6.18 (1.39)	6.6 (2.67)
Wilson B-factor	89.02	72.19	70.89
*R*_merge_	0.0756 (0.5209)	0.101 (0.9234)	0.099 (0.726)
*R*_meas_	0.08273	0.1213	0.104 (0.771)
CC_1/2_	0.99 (0.915)	0.99 (0.418)	0.99 (.760)
CC*	1 (0.978)	0.998 (0.768)	1 (.929)
Number of molecules per asymmetric unit	2	4	2
**Refinement**			
*R*_work_	–	0.2077 (0.3364)	–
*R*_free_	–	0.2509 (0.3591)	–
Number of atoms	–	–	–
Protein	–	2767	–
Ligand/ion (Zn^2+^)	–	4	–
Water	–	5	–
Protein residues	–	363	–
Ramachandran plot	–	–	–
Favoured (%)	–	341 (94)	–
Allowed (%)	–	22 (6)	–
Outliers (%)	–	0 (0)	–
RMS(bonds)	–	0.012	–
RMS(angles)	–	1.18	–
Average B-factor:	–	99.8	–
Protein	–	99.8	–
Ligand/ion (Zn^2+^)	–	137.10	–
Water	–	68.50	–

### Crystal structure refinement and analyses

The experimental phases for the SeMet–ComFB data were determined using AutoSol in the PHENIX package with default options except the ‘autobuild model’ option deselected [22,23]. The resulting heavy atom Se-sites, initial density modified map and experimental data with phases were combined with the ComFB sequence for initial model building using AutoBuild in PHENIX with default options including ‘build SeMet residues’. The resulting model was used for molecular replacement with the native data using Phaser [24]. Model building was subsequently done using Coot [25]. The native structure was refined using PHENIX. Refine in PHENIX with the following refinement strategies: ‘XYZ coordinates’, ‘TLS parameters’ and ‘Individual B-factors’. The following restraints were used: ‘Optimize X-ray/stereochemistry weight’, ‘Optimize X-ray/ADP weight’ and ‘NCS restraints’ (for initial rounds). All restraints were used with the default options. Residues at the N- and C-termini, including uncleaved histidine–tag, were disordered and therefore the final model includes residues 1–91, 1–89, 1–91 and 1–88, for chains A, B, C and D respectively. The dimer formed by chains A and B was used for structural analyses because it had better defined electron density and thus more complete models at the C-termini.

A search for known protein structures with a fold similar to that of ComFB was performed using PDBeFOLD and DALI [26,27]. Analyses of contact surface areas between ComFB protomers in the crystal lattice were done on the PISA server [28]. Figures were generated in PyMOL (Schrödinger, LLC).

### *B. subtilis* transformation efficiency assay

Starter cultures of *B. subtilis* strains in LB (*A*_600_ 0.60–1.25) were diluted at least 200-fold into 1× MC medium and grown for 5 h at 37°C with agitation to induce the competent state. Two microgram pDR110 (David Z. Rudner, unpublished) was added per millilitre of *B. subtilis* culture and the cells were grown for an additional 1–1.5 h at 37°C with agitation. Dilutions were performed as needed in 1× TBase supplemented with 1 mM MgSO_4_ and cells were plated to LB+spectynomycin plates to select transformants and DSM plates to determine cfu. Plates were incubated at 37°C for 20–22 h and colonies were counted.

### Fluorescence microscopy

Microscopy was performed on an Axio Imager.M1 upright microscope equipped with a 100× EC Plan-NEOFLUPR objective (oil, NA 1.3) and filters for GFP and mKate. Images were captured with a charge-coupled device (CCD) camera (Photometrics CoolSNAP HQ^2^). Exposure times varied from 0.5 to 2 s. Images were acquired with AxioVision software (version 4.8). To perform microscopy, the *B. subtilis* cells were grown to the competent state in 1× MC medium, collected by centrifugation (3 min, 6000 ***g***), resuspended in 1× PBS and mounted between glass slide and coverslip.

## RESULTS

### Crystal structure of ComFB

To gain insights into the role ComFB plays in *B. subtilis* cells, we first decided to characterize this protein *in vitro*. We developed a two-step purification protocol for N-terminally 6×His–tagged *B. subtilis* ComFB protein expressed in *E. coli* that yielded highly pure protein (Figure 1C). ComFB eluted at an apparent molecular weight of ∼35 kDa from a Superdex75 size exclusion chromatography (SEC) column, suggesting that the protein is a dimer (Figure 1D). Purified ComFB was crystallized and the structure determined by single-wavelength anomalous diffraction using selenomethionine-substituted protein. The selenomethionine-substituted protein was crystallized in a P22_1_2_1_ spacegroup with two molecules in the asymmetric unit. The final native ComFB structure was obtained after molecular replacement and refinement into a higher resolution [2.48 Å at an I/σ(I) of 1.98] native dataset with P2_1_ symmetry and four molecules in the asymmetric unit (Table 1). The four molecules are arranged as two dimers, each with two-fold symmetry.

**Figure 2:**
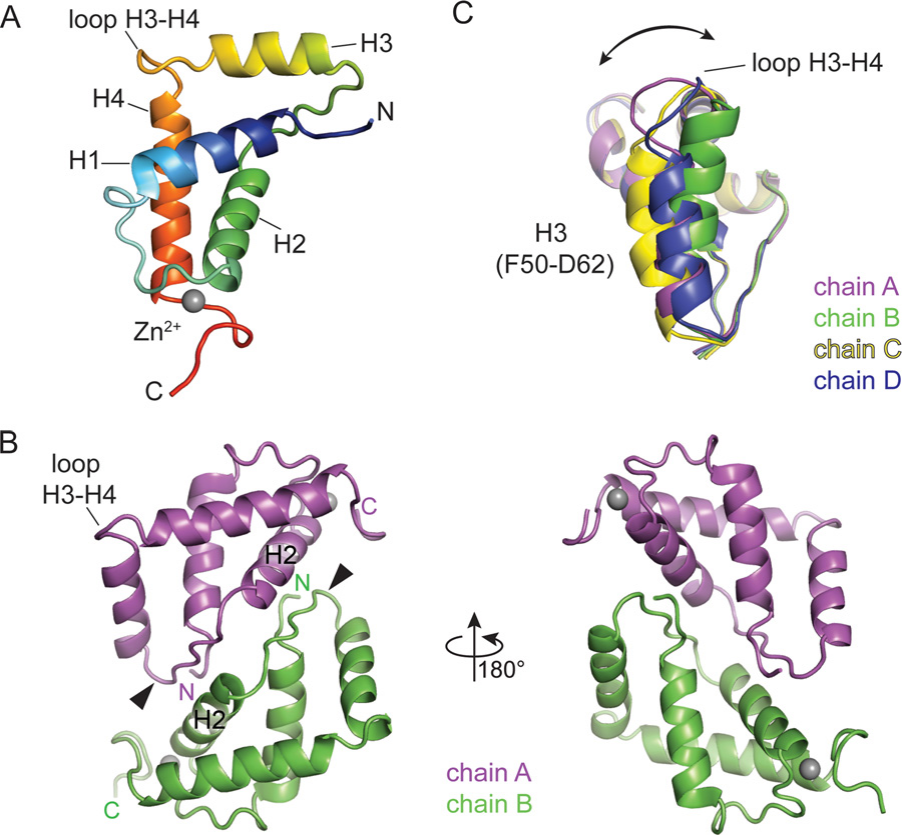
3D structure of ComFB protein (**A**) Monomer of ComFB in cartoon representation showing positions of the four α-helices. (**B**) ComFB dimer (chains A and B) in two different orientations, showing N-terminal tail, β-like stretch (arrowhead) and helix H2 forming the interface. (**C**) Most of the structure variation among the four ComFB molecules in the asymmetric unit originates from the position of helix H3 and the proceeding H3–H4 loop as is illustrated in the present study by superimposing the four molecules using all Cα atoms except those of residues 50–65.

The structure of ComFB consists of four α-helices, H1–4 and a six-residue extended β-strand-like loop between helices H2 and H3 (Figures 1B and 2A). The extended loop, formed by residues 42–47, is wedged between the subunit's N-terminal segment and the α-helix H2 of the adjacent subunit in the dimer (Figure 2B; more on the dimer below). All four molecules in the asymmetric unit are similar to pairwise RMSD values ranging from 1.1 to 1.6 Å over all Cα atoms for residues 1–88. Most of the structure variation originates from the position of helix H3 and the proceeding H3–H4 loop (Figure 2C) and correspondingly the RMSD values drop to 0.3–0.6 Å when omitting residues 50–65 (H3).

The α-helical fold resembles that of several small domains identified by the DALI or PDBeFOLD webservers [26,27]. Examples include the C-terminal domain of poly(A)-binding protein homologue C-terminal domain (PABPC [29]) and ubiquitin-binding CUE domains (UbCUE [30]). Helices H1, H2 and H4 of ComFB can be superimposed with RMSD values of 2–3 Å with these compact folds with three or four α-helices. However, there is no significant sequence conservation between these domain families, the extended loop is absent and the H3 helix is either absent or in a very different position in PABPC and UbCUE (Supplementary Figure S1). Therefore, these weak structural similarities alone are unlikely to provide significant insights into the function of ComFB.

### ComFB binds zinc

**Figure 3:**
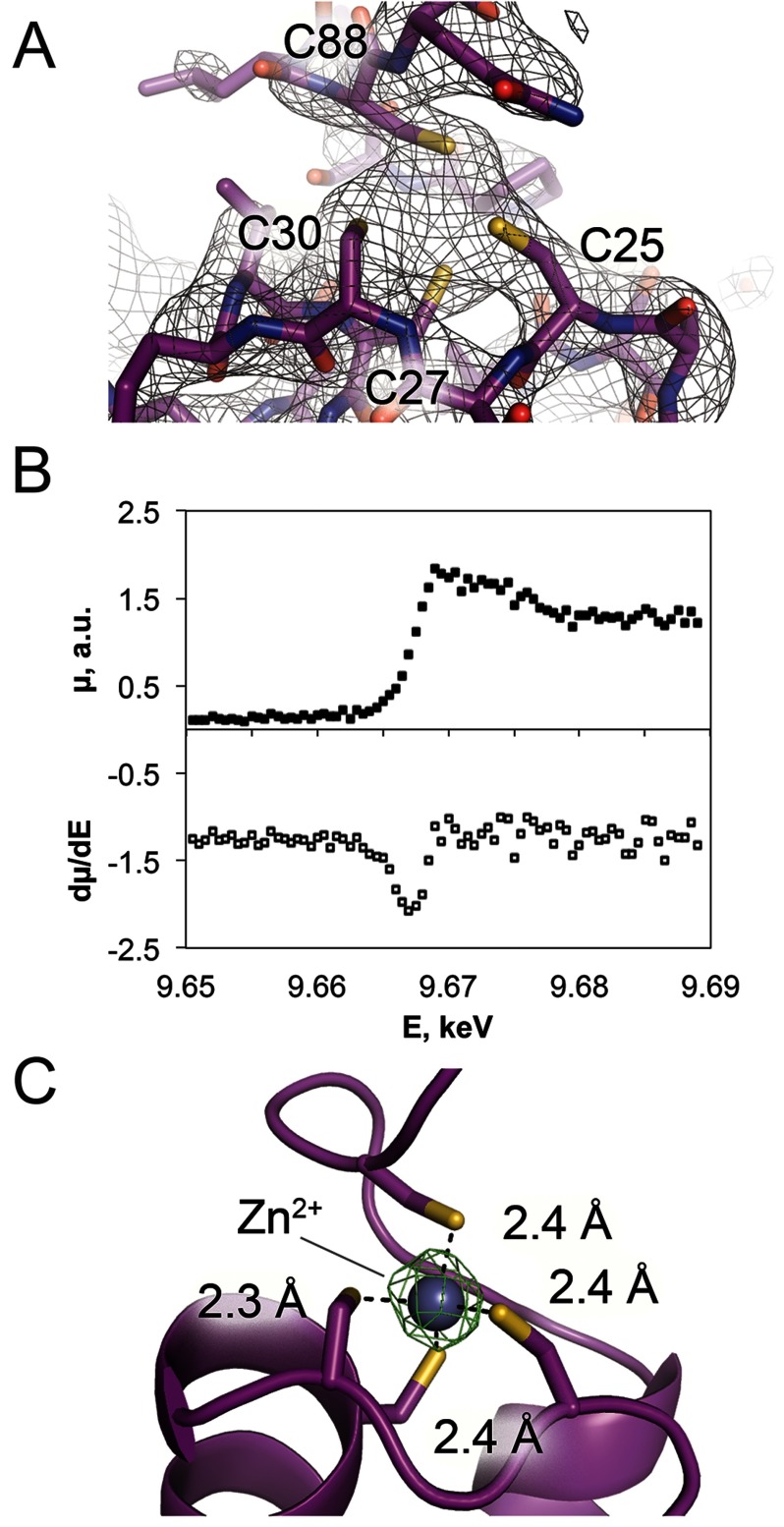
Zinc-binding site of ComFB (**A**) Simulated-annealing omit map contoured at 1.5σ, showing a significant extra density flanked by four cysteine residues of ComFB. (**B**) XAFS spectra of native ComFB crystals that indicate presence of Zn atoms. (**C**) Anomalous difference map (green mesh; 10σ) and refined ComFB structure. The distances from the cysteine sulfur atoms to the Zn^2+^ cation are indicated.

Electron density maps during the refinement showed the presence of extra electron density in a region of the ComFB structure in which all four cysteines converged (Figure 3A). We thus hypothesized that a Zn^2+^ ion was bound to the ComFB protein. We used two experimental tests to confirm the identity of the Zn^2+^ ion. We collected an X-ray absorbance spectrum (XAFS) that showed an edge at 9666.25 eV (1.2827 Å), characteristic for Zn^2+^ ions (Figure 3B), indicating that Zn^2+^ was probably present in the crystal, although no Zn^2+^ was introduced during protein purification or crystallization. We also collected a complete X-ray diffraction dataset on a native ComFB crystal at wavelength of 1.2823 Å to measure anomalous signals. We observed an anomalous difference density peak at the position of the hypothesized Zn^2+^ ion (Figure 3C) in each of the four molecules within the asymmetric unit. These were the only four density peaks observed at this intensity in the anomalous difference map. We therefore conclude that each ComFB molecule coordinates one Zn^2+^ ion.

In ComFB, the Zn^2+^ ion is coordinated in a typical tetrahedral arrangement by four cysteine residues (Figure 3C). Three of the cysteine ligands, C25, C27 and C30, are closely spaced in the primary sequence. Such close spacing of the ligands is characteristic of short zinc-binding loops [31]. In these zinc-binding motifs, as in ComFB, the cysteines are found in loops that are lacking secondary structure organization. In ComFB the fourth cysteine that completes the binding site comes from the C-terminal tail, residue C88 (Figure 3). Similar short Zn^2+^-binding loops with one distant and three closely spaced cysteines were previously found in a few proteins, including DNA/RNA-modifying enzymes such as tRNA–guanine transglucosylase, intron-encoded homing endonuclease I–PpoI and Vsr nuclease [32–34].

An alignment of ComFB homologues shows that the Zn-binding motif is highly conserved, with the fourth position being either cysteine or histidine (Figure 1B). In the ComFB crystal structure, the C-terminal residues beyond C88 are largely disordered, suggesting that C88 binding to the Zn^2+^ ion helps lock helix H4 into place. We propose that the bound Zn^2+^ ion stabilizes the ComFB fold or at least this particular arrangement of helix H4. Thus, the bound Zn^2+^ ion may serve a permanent structural role. Alternatively, the Zn^2+^ ion could play a regulatory by controlling the position of the H4 helix and its accessibility to potential interacting partners. However, we note that the Zn^2+^ ion binding site in ComFB probably has a relatively high affinity and/or a very slow off-rate because we were able to observe the bound Zn^2+^ ion even after extraction and purification of the protein in the absence of additional Zn^2+^.

### ComFB is an obligate dimer

We observed four ComFB molecules in the asymmetric unit in the native P2_1_ crystal form (Figure 2A), whereas our SEC data suggest that ComFB may form dimers in solution (Figure 1D). To identify which, if any, of the observed crystal packing interfaces may correspond to a physiological interface, we analyzed the interfaces using the PISA server [28].

The results yielded two possible types of two-fold symmetric dimer interfaces. One type generates homodimers of chains A and B and C and D respectively, within the asymmetric unit, burying a total of 1550 Å^2^ of surface area (for the A–B dimer), with 11 hydrogen bonds. This dimeric arrangement is built around two-fold symmetric interactions between the extended H2–H3 loop with the helix H2 of the adjacent subunit, as described above (Figure 2C). A second type of two-fold symmetric dimer connects chains B and D through a parallel interface between their H4 helices, burying a total of 1120 Å^2^ of surface area (Supplementary Figure S2). However, neither chain A nor chain C forms an analogous interaction and the side chains on this second interface (V71, A74, E75) are less conserved, based on PFAM family PF10719 alignments and compared with residues on the larger interface. Furthermore, the statistical analyses performed by the PISA webserver suggest that this second interface is unlikely to be stable in solution. We therefore conclude that the first interface type is most likely to represent a possible stable dimer.

In this dimeric arrangement there are several polar interactions present between the neighbouring subunits: the hydroxy of Y46, within the β-stretch of one subunit hydrogen-bonds with both N4 and E7 of the adjacent subunit; D33 in helix H2 of one subunit interacts with T48 at the edge of the β-stretch of another monomer; N40 of helix H2 interacts with the backbone amino group of Y46 of the neighbouring subunit. Alignments of ComFB homologues show that residues N4, E7, D33, N40 and Y46 that are involved in this dimer interface are highly conserved (PF10719; Figure 1B).

**Figure 4:**
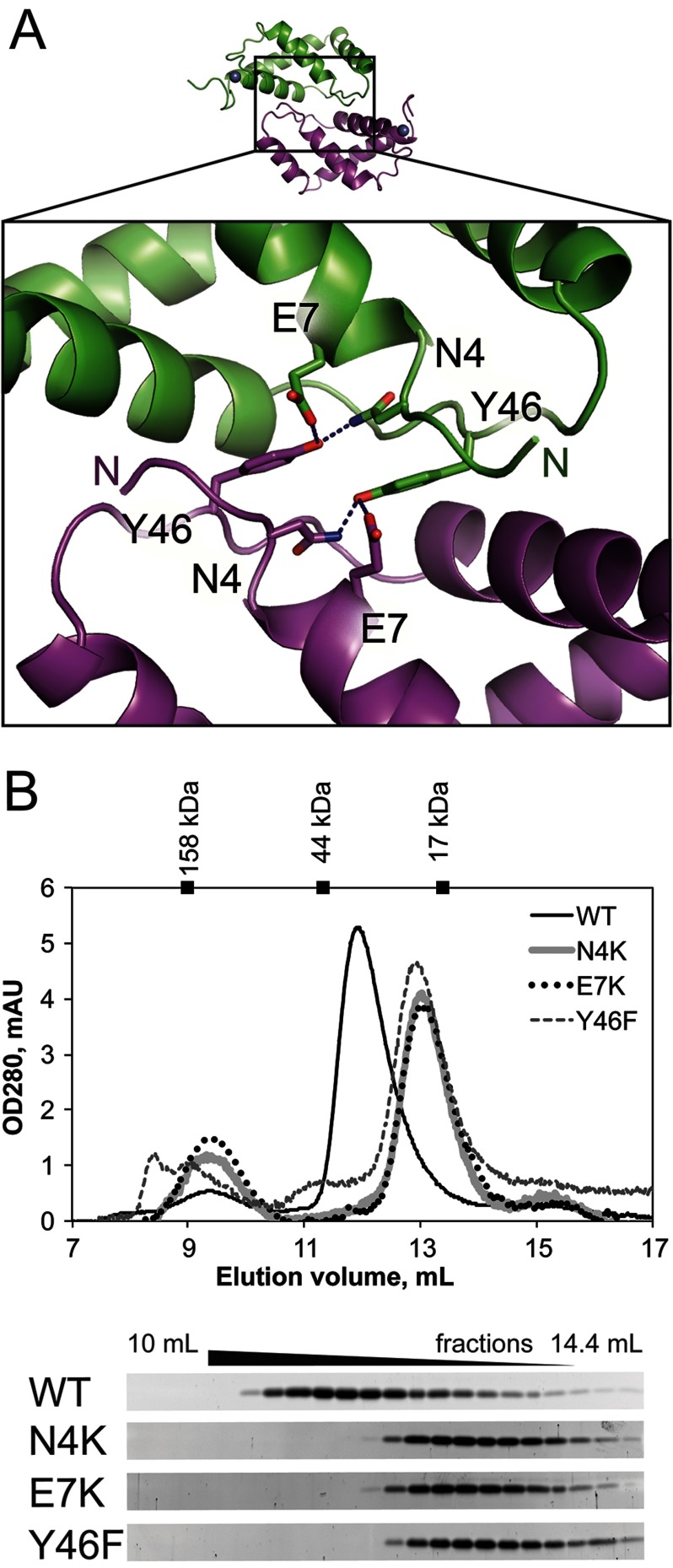
Dimerization interface of ComFB (**A**) Polar interactions at the dimer interface with a buried surface area of ∼1550 Å^2^. (**B**) SEC analyses of mutant ComFB proteins show that all three tested variants differ from wild-type protein. Bottom of the panel shows SYPRO Ruby stained SDS-PAGE gels of the fractions collected during SEC runs that identify ComFB as a major species in each of the preparations.

To determine whether the dimer identified in the crystal (Figures 2B and 4A) corresponds to the apparent dimer observed by SEC, we introduced various single amino acid mutations that should disrupt the ComFB dimer interface: N4K, E7K or Y46F. Each protein variant was expressed and purified. The yields of soluble protein were significantly lower than those we observed for wild-type ComFB, already hinting at the importance of these residues. We performed SEC experiments on Superdex75 using samples of wild-type and ComFB variants at similar concentrations, to compare the behaviour of these proteins in solution. All three tested variants, N4K, E7K or Y46F, eluted later than the wild-type protein with a ∼1 ml shift in the elution volume (Figure 4B). This shift in the elution volume is consistent with the interface-disrupting mutations yielding monomeric ComFB. These solution-based experiments therefore confirm that the largest contact interface observed in the ComFB crystal structure does indeed represent a dimerization interface of the ComFB protein in solution. We conclude that ComFB is a stable, obligate dimer under physiological buffer conditions.

### ComFB does not contribute to competence development under standard laboratory conditions

**Figure 5:**
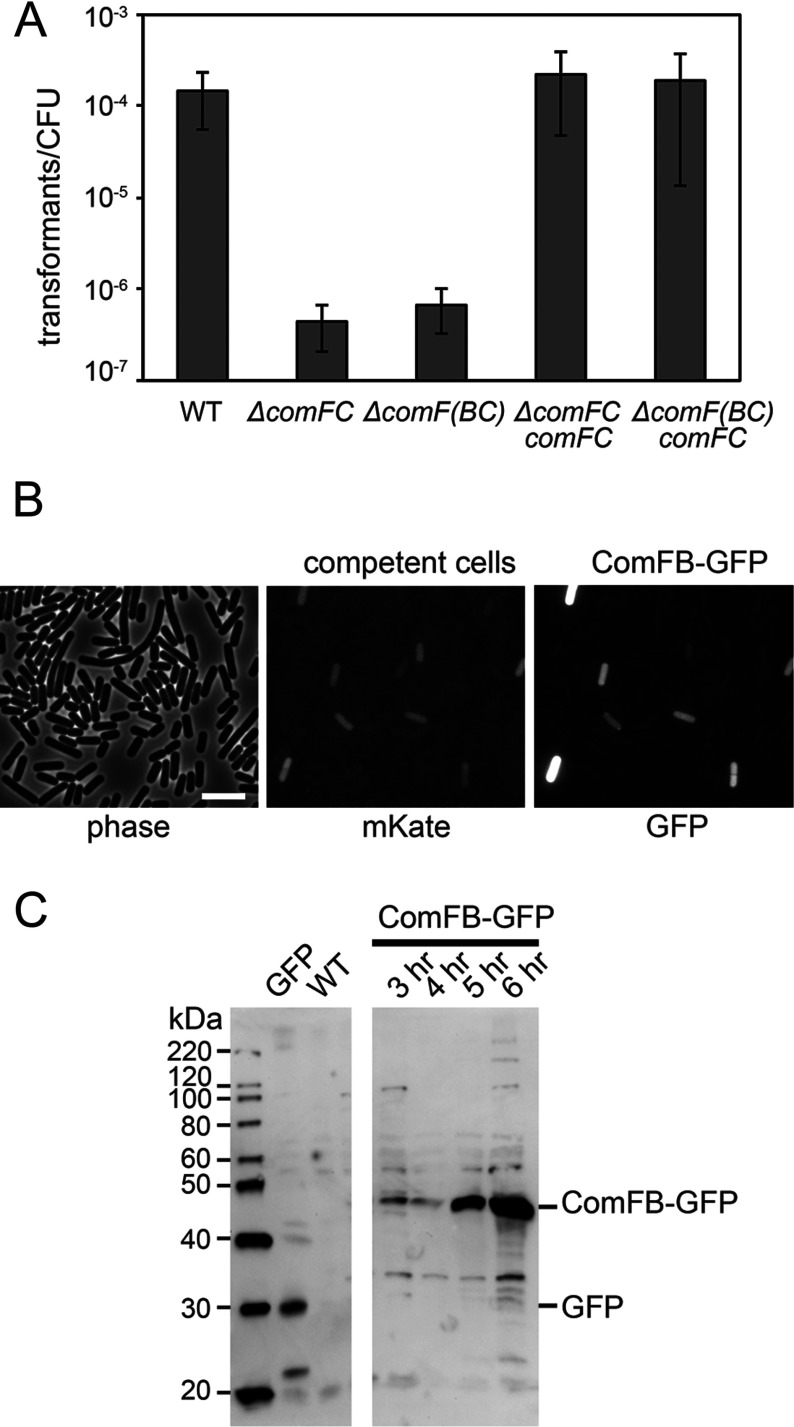
ComFB is expressed upon development of competence in *B. subtilis* although its absence does not affect transformation efficiency (**A**) Deletion of *comFB* does not significantly affect transformation efficiency of *B. subtilis* cells. Strains deleted for *comFC* or *comF(BC)* were complemented with *comFC* under the *comFA* promoter and tested in transformation efficiency assays. WT is the wild-type PY79 *B. subtilis* strain. The average transformants/cfu for three experiments is plotted. Error bars indicate ±1 S.D. (**B**) ComFB–GFP and *P_comK_*-driven mKate co-expression. A *lacA::P_comK_-mKate* construct was introduced to a strain with a *ycgO::P_comFA_–comFB–gfp* background. Cells were grown to competence in 1× MC media and examined by fluorescence microscopy. ComFB–GFP expression is highly correlated with mKate expression, verifying that only competent cells express ComFB–GFP. Scale bar is 10 μm. (**C**) ComFB–GFP expression. Strains carrying *ycgO::P_comFA_–comFB–gfp* were grown in 1× MC media and normalized samples were harvested at specified time points. Western blots of the samples, probed with α-GFP antibodies, verify that ComFB–GFP is detectable beginning around 4–5 h time points, corresponding with the onset of competence. The location of free GFP is indicated and confirms that the levels of cleavage products are low. A strain carrying an *amyE::P_veg_–gfp* construct was used as a positive control for free GFP, whereas wild-type (PY79) strain served as a negative control.

The original work on the function of *comF* operon in DNA uptake revealed that a transposon insertion in *comFB* resulted in a 10-fold decrease in transformation efficiency and that was attributed to a polar effect on *comFC* expression [7]. We constructed a set of strains to investigate the contributions of *comFB* and *comFC* to transformation efficiency. Deletion of both the *comFB* and the *comFC* genes together results in a significant drop in competence of *B. subtilis* which is consistent with previously reported results (Figure 5A). However, in our experiments, deletion of *comFB–comFC* or *comFC* alone resulted in a 300-fold decrease in transformation efficiency that is about 30 times larger than the previously observed effect. Ectopic expression of *comFC* rescues the defect in transformation efficiency to wild-type levels. Our results therefore indicate that ComFB is unlikely to affect transformation under standard tested conditions; this is consistent with previously reported data.

### ComFB is expressed in competent *B. subtilis* cells

Late competence genes are known to have a specific temporal and spatial distribution [12–14]. To confirm that ComFB is produced when expected based on its gene's presence in a late competence operon and to analyze its spatial distribution, we expressed ComFB fused to the GFP from the *comFA* promoter. We ectopically expressed this fusion in a *B. subtilis* strain that also expressed a red fluorescent protein (mKate) under the control of promoter of major competence transcriptional regulator ComK. Strains containing both constructs were grown to competence and analyzed by fluorescence microscopy. The results show that ComFB is produced only in the subset of cells that are also expressing a significant amount of mKate (Figure 5B).

The microscopic analyses revealed that ComFB exhibits diffuse cytosolic localization which is consistent with the prediction that it is a soluble cytoplasmic protein (Figure 5B). To rule out possible proteolysis of the ComFB–GFP fusion, we performed Western blot analysis, which confirmed that there is no significant accumulation of free GFP in the tested strains (Figure 5C), suggesting that ComFB–GFP is in fact cytosolic. Taken together with the transformation assays, these results suggest it is unlikely that ComFB forms a part of the DNA uptake apparatus.

## DISCUSSION

In the present study, we characterized the late competence protein ComFB from *B. subtilis in vitro* and *in vivo*. We determined its 3D structure, showing a compact fold of four α-helices connected by long loops including one in a β-strand-like conformation. The ComFB fold resembles the all-helical folds of multiple proteins including several DNA-modifying enzymes and regulatory proteins, although there is little sequence conservation. ComFB forms a stable dimer in solution and structure-guided mutagenesis revealed that it is likely an obligate dimer with a highly conserved interface. Each subunit of the ComFB dimer tightly binds a Zn^2+^ ion using four cysteine ligands.

Earlier it was proposed that ComFB forms part of the DNA uptake apparatus, as ComFB–YFP fusion was localized to the cellular poles when expressed under a xylose-inducible promoter [14]. When we expressed ComFB–GFP fusion under its native *comFA*-promoter we observed a diffuse cytosolic staining indicative of a soluble protein and no detectable enrichment at poles. It is consistent with a previous report [7] and our data that deletion of ComFB does not significantly affect development of the competent state and probably does not contribute to the DNA uptake machinery that might be localizing to the poles under tested conditions.

It is interesting to consider that the *comFB* ORF, when present, usually overlaps with the following *comFC* ORF for a few codons (Figure 1A). Such an overlap of ORFs in bacteria is often a way to translationally couple two polypeptides that might form a complex or regulate each other's activity [35].

**Figure 6:**
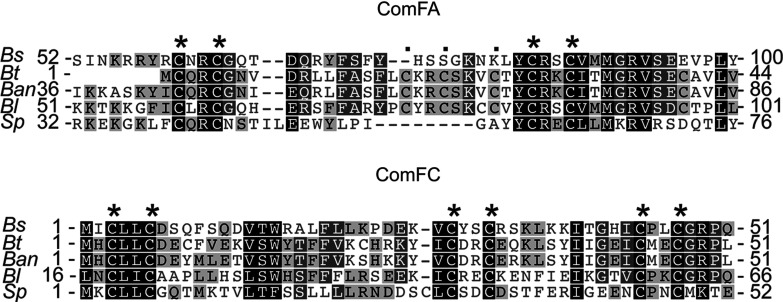
Cysteine motifs in proteins of comF operon Fragments of ComFA and ComFC alignments illustrating presence of classic four-cysteine motifs in ComFA and cysteine-rich N-terminus of ComFC protein. *Ban, Bacillus anthracis; Bl*, *Bacillus lichenomorphis*; *Bs*, *Bacillus subtilis*; *Bt*, *Bacillus thuringiensis; Sp*, *Streptococcus pneumoniae*.

It is also intriguing to consider that the other two proteins encoded by the *comF* operon, ComFA and ComFC, contain clearly recognizable polycysteine motifs (Figure 6). The ComFA ATPase has a tetracysteine motif and metal binding is one of the characteristic features of some DEAD-box helicases. ComFC possesses a cysteine-rich N terminus. Therefore, all three proteins encoded in the *comF* operon, including ComFB, as shown in the present work, probably bind metal.

Zinc is an oxidation-neutral metal that often protects cysteines from oxidation or serves as a sensor of the cysteine oxidation state [36]. An example of this behaviour is found in the anti-sigma factor RsrA, which controls the activation of the disulfide stress-specific sigma factor SigR. Zinc-bound RsrA forms a stable complex with SigR thus inhibiting the activity of the latter. Under oxidative stress conditions, cysteines in RsrA are oxidized to form a disulfide bond, thus expelling the bound Zn^2+^ ion. Zinc loss in turn leads to dissociation of the RsrA from the complex leading to release and activation of the SigR factor [37,38].

The *comF* operon was specifically down-regulated in the absence of the high affinity zinc transporter ZnuABC [15]. Another zinc transporter, ZosA, is under strict control of the oxidation stress response regulator PerR and is induced in the presence of hydrogen peroxide. The connection between competence development, oxidative conditions and zinc levels is unclear. However, it is possible that ComF proteins may be involved in sensing either oxidative stress or zinc levels or both and relaying this information to affect competence development.

The newly identified zinc-binding site in ComFB uses a fourth ligand that is quite distant from the other three in the primary sequence, which could provide increased accessibility in comparison with more canonical zinc-binding sites. The N terminus appears to be locked by the dimerization interface contacts, whereas the C terminus is stabilized by zinc binding (Figures 2A and 3A). Moreover, the long loop between H1 and H2 forms the metal-binding site (Figure 3C), whereas the loop between H3 and H4 is more flexible and shows a lot of variability in the crystal structure (Figure 2C). H2 contacts the extended β-like stretch on one end and participates in the zinc-binding site on the other end. These connections lead us to hypothesize that dimerization and zinc binding might depend on each other.

It will be interesting to investigate whether ComFB may serve as a ‘sensor’ of zinc concentration or cellular oxidative state. If true, ComFB might participate in a regulatory process linked to the same regulatory network as PerR, ZosA, ZnuABC. Since our *in vivo* experiments showed that ComFB does not constitute part of the DNA uptake machinery under the tested conditions, we hypothesize that this protein may play a regulatory role in competence development by sensing zinc levels or oxidative stress with its zinc-binding site. Further experiments will be required to establish the precise function of this competence-related protein.

## Online data

Supplementary data
