# Supplementary material for: Structural characterization of the late competence protein ComFB from Bacillus subtilis
Source: Biosci Rep. 2015 Mar 31;35(2):e00183. doi: 10.1042/BSR20140174 (PMC4381287; doi:10.1042/BSR20140174)
Supplement: Supplementary data [file bsr035e183ntsadd.pdf]

Supplemental Materials

Plasmid Construction

pDX001 [pET28b-*His6-comFB*] The *comFB* sequence (oDX001/oDX002; NheI/BamHI) was ligated into pET28b (Novagen).

pDX010 [Δ*comFC::cat*] Fragments from upstream (oDX023/oDX024; XbaI/SphI) and downstream (oDX025/oDX026; Sal/EagI) of *comFC* were ligated into pSC009 flanking *cat*. pSC009 was made by site-directed mutagenesis of pKM074 (K.A. Marquis & D.Z. Rudner, unpublished) to remove the AvaII sites.

pDX017 [*ycgO::PcomFA-comFB-gfp (kan)*] The NdeI site in *comFB* was removed by site-directed mutagenesis of pDX001 with oDX036. *comFB* from the resulting plasmid (oDX034/oDX008; NdeI/XhoI) was ligated into pSC029 to generate pDX016, containing *comFB* fused to *gfpmut2* downstream of *PcomFA*. pSC029 contains a *PcomFA* fragment (oSC014/oSC073; EcoRI/XhoI) ligated into pKL147[1], replacing the *dnaX* fragment upstream of *gfpmut2*. The *PcomFA-comFB-gfpmut2* fragment from pDX016 (EcoRI/BamHI) was ligated into pKM152 (K.A. Marquis & D.Z. Rudner, unpublished) to generate pDX017.

pDX023 [*lacA::PcomK-mKate (erm)*] A *PcomK* fragment (oDX045/oDX044; SalI/BamHI) was ligated into pUK19[2], generating pDX021. A *mKate2* fragment (oDX041/0DX042; XhoI/BamHI) from pTMN387[3] was ligated into pDX021, generating pDX022. oDX041 includes an rbs sequence for *mKate2*. The *PcomK-rbs-mKate2* fragment (SalI/BamHI) from pDX021 was subcloned into pDR183 (D.Z. Rudner, unpublished).

pDX028 [Δ*comF(BC)::cat*] A fragment from upstream of *comFB* (oDX015/oDX016; EcoRI/SphI) was ligated into a pSC009-derivative. The fragment was then subcloned from the resulting plasmid into pDX010 by XbaI/BamHI, replacing the sequence from upstream of *comFC*.

pDX029 [*ycgO::PcomFA-comFC (kan)*] The *comFC* sequence (oDX004/oDX005; NdeI/BamHI) was ligated into pET28b (Novagen) and then subcloned into pDX017 by NdeI/BamHI, replacing *comFB-gfp*.

pVP038 [*amyE::Pveg-gfp(spec)*] *Pveg* (oVP041/oVP042; EcoRI/HindIII) was ligated into pMF23[4] replacing *PspoIID* upstream of *gfp*.

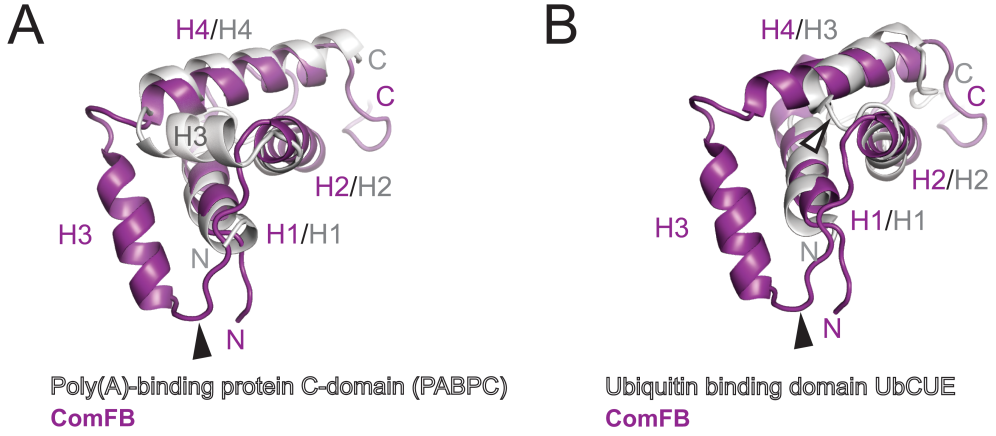

Figure S1. *ComFB fold resembles several known helical folds*. (A) Overlay of a ComFB subunit (purple) with the human hyperplastic discs protein, homolog of C-terminal domain of poly(A) binding protein (PABPC domain, 1i2t; light grey). (B) Overlay of a ComFB subunit (purple) with the ubiquitin binding domain CUE (UbCUE, 2lvo; light grey). α-helices of the structures are indicated. Solid triangle highlights an extended β-strand-like stretch between H2 and H3 of ComFB that in combination with a long H3 helix makes ComFB fold different from both PABPC and UbCUE folds that have either short H2-H3 loop and short H3 helix (PABPC), or missing both of these elements (UbCUE; indicated with an open triangle).

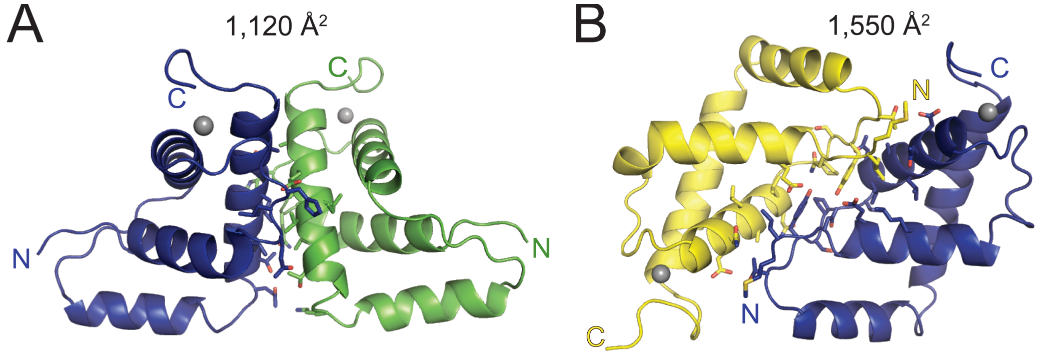

Figure S2. *Two dimeric ensembles present in the ComFB crystal as found by PISA server*. (A) Dimer of chains B and D. (B) Dimer of chains C and D. A/B dimer is highly similar and presented in Figure 2B. Coloring as in Figure 2. Residues that are 4 Å or less from the neighboring subunit are shown as sticks. Estimated contact surface areas are indicated above the respective ensembles. Bound zinc cation is presented as grey spheres.

Tables

Table S1. Bacterial strains used in this study.

Strain name	Genotype	Source
E. coli		
BL21(DE3)	*fhuA2 [lon] ompT gal (λ DE3) [dcm] ΔhsdS*<br>*λ DE3 = λ sBamHIo ΔEcoRI-B*<br>*int::(lacI::PlacUV5::T7 gene1) i21 Δnin5*	
B. subtilis		
PY79		Laboratory background strain
bDX011	*ΔcomFC::cat*	This work
bDX029	*ΔcomF(BC)::cat*	This work
bDX031	*ΔcomFC::cat; ycgO::PcomFA-comFC(kan)*	This work
bDX032	*ΔcomF(BC)::cat; ycgO::PcomFA-comFC(kan)*	This work
bVP047	*amyE::Pveg-gfp(spec)*	This work
bDX010	*ycgo::PcomFA-comFB-gfp(kan)*	This work
bDX016	*lacA::PcomK-mKate(erm)*	This work
bDX017	*ycgO::PcomFA-comFB-gfp(kan); lacA::PcomK-mKate(erm)*	This work

Table S2. Oligonucleotides used in this study.

Oligo-nucleotide	Sequence (5'->3')	Purpose
oDX001	ctagctagcatgcttgtcaattcaaaagaaatcg	pET28b-*His6-comFB*
oDX002	cgcggatcctcagtttatgaatgcctcctcctg	pET28b-*His6-comFB*
oDX004	cgcggatccttagcttctgatcaaggtaaaag	*ycgO::PcomFA-comFC (kan)*
oDX005	gggaattccatatgatctgtttattatgtgattcgcaa	*ycgO::PcomFA-comFC (kan)*
oDX008	ccgctcgaggtttatgaatgcctcctcctgcttc	*ycgO::PcomFA-comFB-gfp (kan)*
oDX015	acatgcatgcgtgaaaagatggatagagtttcatg	*ΔcomF(BC)::cat*
oDX016	ggaattcttttacagctcctcatcatcgg	*ΔcomF(BC)::cat*
oDX023	gctctagagcataaagaatatgcggacg	*ΔcomFC::cat*
oDX024	acatgcatgccataataaacagatcagtttatgaatgcc	*ΔcomFC::cat*
oDX025	acgcgtcgacatgattctgtttttatgccg	*ΔcomFC::cat* and *ΔcomF(BC)::cat*
oDX026	actgtacggccgtagggaatcagctgtttttgg	*ΔcomFC::cat* and *ΔcomF(BC)::cat*
oDX034	ggaattccatatgcttgtcaattcaaaagaaatc	*ycgO::PcomFA-comFB-gfp (kan)*
oDX036	cgaccgatatatggaccagcttcacatggcctgtacttgccaagtatgc	*ycgO::PcomFA-comFB-gfp (kan)*
oDX041	ccgctcgaggatggaggccataatatggattcaatagaaaaggtaagcg	*lacA::PcomK-mKate(erm)*
oDX042	cgcggatcc ttatctgtgcccc	*lacA::PcomK-mKate(erm)*
oDX044	tcggatccctcgagctgcaaaatttatactaataatctatcatc	*lacA::PcomK-mKate(erm)*
oDX045	agacgtcgacggcggaacaattgtgaacgg	*lacA::PcomK-mKate(erm)*
oSC014	ggaattccaaatctccgtttttagagcggagattttttatattctta	*ycgO::PcomFA-comFB-gfp (kan)*
oSC073	ccgctcgagcggaaatccatatggcacgcctcctttcgaaacagtatg	*ycgO::PcomFA-comFB-gfp (kan)*
oTS387	tcattacgatttcttttgatttgacaagcatgctagccatatgg	N4K
oTS388	ccatatggctagcatgcttgtcaaatcaaaagaaatcgtaatga	N4K
oTS397	cagttctttcattacgattttttttgaattgacaagcatgctagcca	E30K
oTS398	tggctagcatgcttgtcaattcaaaaaaaatcgtaatgaaagaactg	E30K
oTS419	gcgatctttttaaaatcagtaacaaaggatggactcactttgtttaaag	Y69F
oTS420	ctttaaacaaagtgagtccatcctttgttactgattttaaaaagatcgc	Y69F
oVP041	agtatgaattcgcggaacataattgaggaatcatag	*amyE::Pveg-gfp(spec)*
oVP042	tagtataagcttacatttattgtacaacacgagccc	*amyE::Pveg-gfp(spec)*

References:

[1] Lemon, K. P., and Grossman, A. D. (1998) Localization of bacterial DNA polymerase: evidence for a factory model of replication, *Science 282*, 1516-1519.

[2] Ju, J., Luo, T., and Haldenwang, W. G. (1998) Forespore expression and processing of the SigE transcription factor in wild-type and mutant Bacillus subtilis, *Journal of bacteriology 180*, 1673-1681.

[3] Norman, T. M., Lord, N. D., Paulsson, J., and Losick, R. (2013) Memory and modularity in cell-fate decision making, *Nature 503*, 481-486.

[4] Fujita, M., and Losick, R. (2003) The master regulator for entry into sporulation in Bacillus subtilis becomes a cell-specific transcription factor after asymmetric division, *Genes & development 17*, 1166-1174.
